# Wild-type and mutant p53 differentially modulate miR-124/iASPP feedback following pohotodynamic therapy in human colon cancer cell line

**DOI:** 10.1038/cddis.2017.477

**Published:** 2017-10-12

**Authors:** Kuijie Liu, Weidong Chen, Sanlin Lei, Li Xiong, Hua Zhao, Dong Liang, Zhendong Lei, Nanjiang Zhou, Hongliang Yao, Ying Liang

**Affiliations:** 1 Department of General Surgery, The Second Xiangya Hospital, Central South University, Changsha, Hunan 410011, China; 2 The People’s Hospital of Zhengzhou University (Henan Provincial People’s Hospital), Zhengzhou 450003, China; 3 Department of Food Science and Engineering, Central South University of Forestry and Technology, Changsha, Hunan 410004, China

## Abstract

Colorectal cancer (CRC) is a most common digestive system malignant tumor. p53 mutation has essential role in cancers and is frequently observed in CRC and presents a huge challenge. p53 mutation has been reported to attenuate the inhibitory effect of photofrin-based photodynamic therapy (PDT). p53 mutation-induced gain of function brings up the dysfunction of carcinogenic factors, including miRNAs. Our research found that PDT suppressed CRC cell viability, reduced the tumor size and prolonged the survival time, all of which could be attenuated by p53 mutation or deletion. After p53 mutation or deletion, several miRNA expression levels were downregulated, among which miR-124 was the most strongly downregulated, whereas iASPP expression was upregulated. p53 binds to the promoter of miR-124 to promote its expression and then inhibited iASPP expression, so as to amplify the inhibitory effect of PDT on wild-type p53 cells. In p53-mutant or -deleted cells, this binding no longer worked to promote miR-124 expression, and iASPP expression increased, finally resulted in promoted CRC cell viability upon PDT. The interactive modulation among miR and iASPP in p53-mutant or -deleted cells may serve as a crucial pathway, which mediates therapy resistance when p53 is mutated or deleted, in the process of PDT treatment of CRC.

In 1997, photodynamic therapy (PDT) was newly classified as a fundamental method for treating tumors by Food and Drug Administration in United States of America, in addition to previously approved surgery, radiotherapy, chemotherapy and biochemical immunotherapy.^1, 2, 3^ It has been identified as one of the prime choices for advanced-stage esophageal cancer along with stenting by National Comprehensive Cancer Network. As for colorectal cancers (CRCs), PDT has also gained increasing attention for its efficacy in advanced cases.^4, 5, 6^ Although PDT has been more and more frequently applied in colon cancer treatment, unexpected challenges also arise, among which p53 mutation presented to be the most severe one.

p53 mutation can be commonly seen in malignancies, especially when patients are found to show resistance to chemotherapy or radiotherapy.^7, 8, 9^ Bond *et al.*^10^ revealed that p53 mutation is common in microsatellite stable, BRAF mutant CRCs. The p53 gene mutation is 0% (0/25), 44.8%(1 (/38), 43.6% (34/78) and 42.1% (8/19), respectively, in normal mucosa tissue, colorectal adenomas, single lesion and multiple lesion of primary colorectal carcinomas,^11^ indicating that the mutation of p53-suppressor gene has a significant role in the procedure of colorectal tumorigenesis. In addition, it has been observed that tumors that bear a mutated p53 may be less responsive to PDT, indicating that p53 and its subsequent pathway may be well involved in terms of PDT efficacy and resistance.^12^ Fisher *et al.*^13^ revealed that wild-type p53 cells are significantly more sensitive to photofrin-mediated PDT. The effect of PDT on tumors is p53 independent. Therefore, by performing more investigation into the role of p53 in PDT treatment, especially the underlying signaling when p53 is mutated, we may further improve its efficacy.

Inhibitor of apoptosis-stimulating protein of p53 (iASPP), the oncoprotein inhibitory member of the ASPP family, could be inhibited so as to affect the CRC cell proliferation according to our previous study.^14^ In certain malignant cells, p53 mutation may trigger the activation of gain of function (GOF), bringing up the expression of some carcinogenic factors while knocking down other tumor suppressors.^15^ Some of these are transcriptional regulators of great importance in regulating tumor growth and cell apoptosis, such as miR-124. In fact, miR-124 has been repeatedly found involved in proliferation and apoptosis of cancerous cells, including CRC.^16, 17^ The mutation of p53 may largely inhibit the expression of miR-124. So a question arises whether the interaction between p53 and miR-124/iASPP has a role in the process of PDT-induced cell apoptosis or when cancer cells show resistance to PDT therapy in p53^mut^ CRC.

Once p53 is mutated or deleted, the expression level of miR-124 was downregulated, whereas iASPP expression was upregulated. After p53 mutation or deletion in CRC cells, we found that the viability of CRC cells was upregulated. When PDT treatment was conducted, the p53^mut^ or p53^−/−^ cells possess stronger viability than that of the p53^wt^ or p53^+/+^ cells. These findings revealed a novel perspective for the investigation of the p53’s role in PDT efficacy and may lead to innovation of PDT by improving photosensitivity.

## Results

### The effect of PDT on cell viability, tumor size and survival percentage

**Figure 1:**
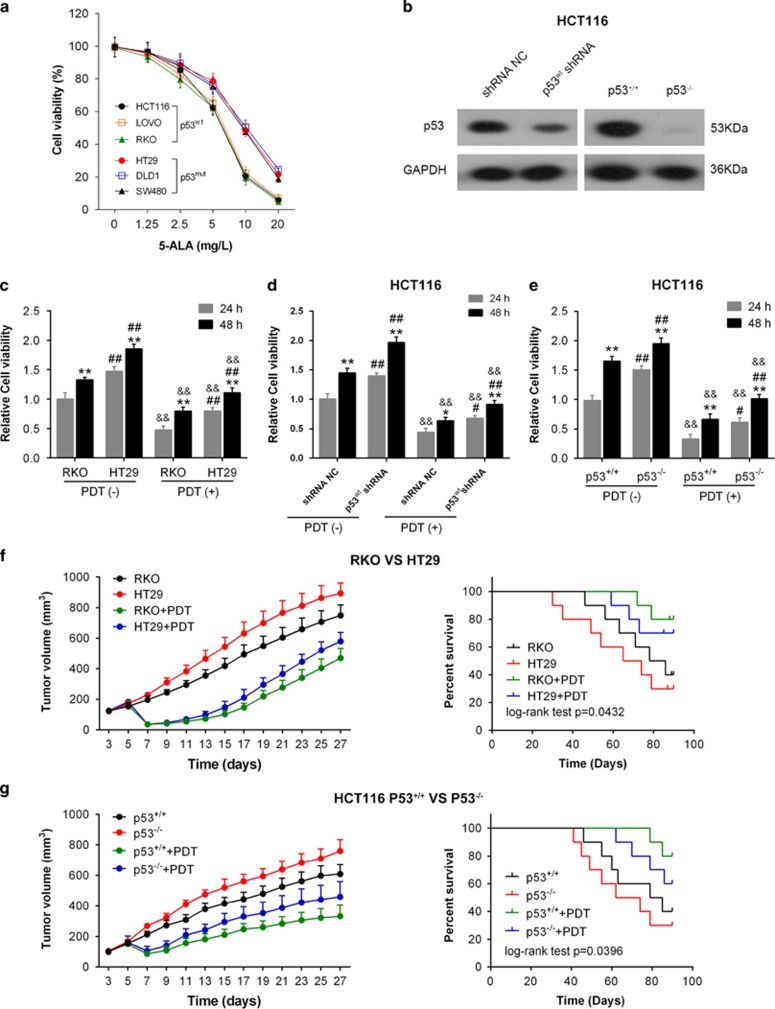
The effect of PDT on cell viability, tumor size and survival percentage. (**a**) The viability of all CRC cells in response to PDT treatment in a concentration-dependent manner was determined by using CCK-8 assays. (**b**) The p53 protein levels in shRNA NC- or p53wt shRNA-transfected HCT116 cells and p53^+/+^/p53^−/−^ HCT116 cells were determined using western blotting assays. (**b**) The viability of p53^wt^ RKO and p53^mut^ HT29 cells was determined at 24 and 48 h in the presence or absence of PDT treatment by using CCK-8 assays. (**c**) HCT116 cells were transfected with shRNA NC or p53wt shRNA. Then the cell viability was determined at 24 and 48 h in the presence or absence of PDT treatment by using CCK-8 assays. (**d**) The viability of p53^+/+^/p53^−/−^ HCT116 cells was determined at 24 and 48 h in the presence or absence of PDT treatment by using CCK-8 assays. (**e**) Tumor volumes of tumors derived from p53^wt^ RKO or p53^mut^ HT29 cells in the absence or presence of PDT treatment were determined every 2 days from day 3 to day 27. The survival analysis was performed to analyze the survival percentage of the mice in the indicated groups. (**f**) Similar assays were performed to analyze p53^+/+^/p53^−/−^ HCT116 cell-derived tumor mice. The data are presented as mean±S.D. of three independent experiments. *P<0.01, 48 h group *versus* 24 h group; #*P*<0.05, ##*P*<0.01, HT29 group *versus* RKO group or p53wt shRNA group *versus* shRNA NC group or p53^−/−^ HCT116 group *versus* p53^+/+^ HCT116 group; &&*P*<0.01, PDT (+) group *versus* PDT (−) group

To investigate the effect of PDT on the viability of p53^wt^ and p53^mut^ CRC cells, we determined the cell viability of HCT116, LoVo and RKO (p53^wt^) and HT29, DLD1 and SW480 (p53^mut^) in response to PDT under a series of concentrations of photosensitizer (5-ALA) (0, 1.25, 2.5, 5, 10, 20 mg/l). Results showed that the viability of all CRC cells was inhibited in response to PDT treatment in a concentration-dependent manner. Moreover, the viability of p53^wt^ cells was inhibited more strongly, compared with the p53^mut^ cells (Figure 1a). Next we determined the viability of p53^wt^ (RKO) and p53^mut^ (HT29) at 24 and 48 h in the absence or presence of PDT treatment. PDT treatment significantly suppressed the cell viability of RKO and HT29 cells, compared with PDT (−) group (&&*P*<0.01). Besides the cell viability of the p53^mut^ CRC cell line HT29 was always much higher than that of the p53^wt^ RKO, either in the absence or presence of PDT treatment (##*P*<0.01; Figure 1c). Then we transfected HCT116 cells with p53^wt^ shRNA or shRNA NC (negative control) in the presence or absence of PDT treatment and verified the p53 protein levels in the indicated cell lines using western blotting assays. Results showed that p53 protein was significantly reduced in p53^wt^ shRNA-transfected HCT116 cells and p53^−/−^ HCT116 cells (Figure 1b). Next, we determined the cell viability by using Cell Counting Kit-8 (CCK-8) assays to evaluate the effect of p53^wt^ knockdown on cell viability. Results showed that PDT treatment significantly suppressed the viability of HCT116 cells in all groups (&&*P*<0.01); however, the cell viability of p53^wt^ shRNA-transfected cells was less strongly suppressed (#*P*<0.05, ##*P*<0.05) (Figure 1d). Similar results were observed in p53^+/+^ and p53^−/−^ HCT116 cells: the viability of all CRC cells was suppressed by PDT treatment (&&*P*<0.01), and the viability of p53^−/−^ cells was suppressed less strongly (#*P*<0.05, ##*P*<0.05) (Figure 1e).

Then the volumes of the tumor derived from RKO (p53^wt^) of HT29 (p53^mut^) cell were measured from day 3 to day 27 every 2 days. Results showed that the tumor volumes without PDT treatment were increased, while the tumor volumes were reduced by PDT treatment on day 7 and slowly increased at the later time points (Figures 1f and g). In addition, the tumor volumes of p53^mut^ and p53^−/−^ cells origin were increased more strongly compared with those of the p53^wt^ and p53^+/+^ cells (Figures 1f and g). Results of the survival analysis showed that the survival percent of the RKO (p53^wt^)+PDT group was the highest, while the HT29 (p53^mut^) group possessed the lowest survival rate (Figures 1f and g). Similar results were observed in p53^+/+^ or p53^−/−^ HCT116 cell-derived tumors (Figures 1f and g). The data suggested that p53 mutation or knockout could promote the CRC cell viability and reduce the sensitivity of CRC cells to PDT treatment.

### Screening and verification of candidate miRNAs for p53

**Figure 2:**
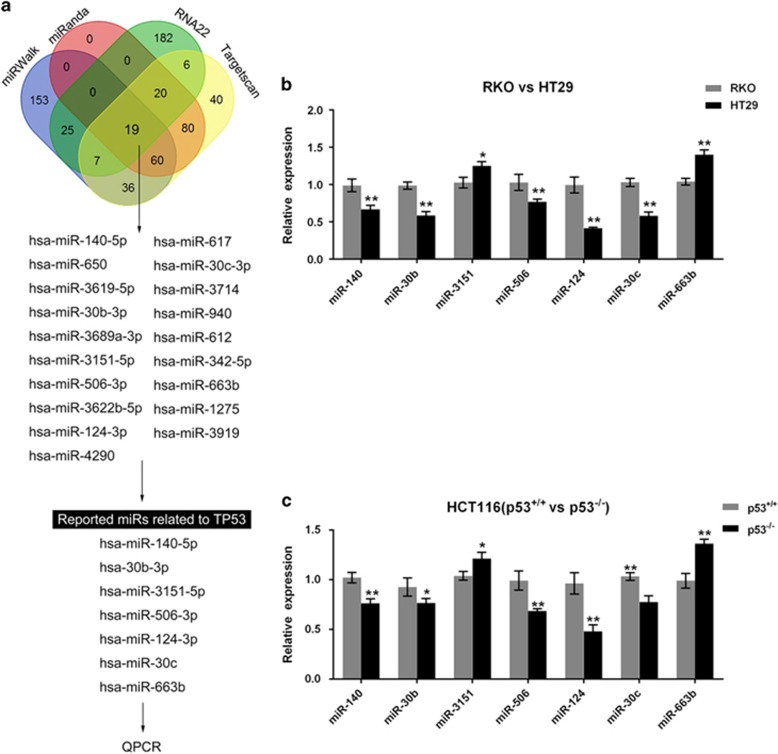
Screening and verification of candidate miRNAs for p53. (**a**) Online tools, including miRWalk, miRanda, RNA22 and Targetscan, were used to screen out candidate miRNAs that could be regulated by p53. (**b**) The expression levels of candidate miRNAs were determined in RKO and HT29 cells by using real-time PCR assays. (**c**) The expression levels of candidate miRNAs were determined in p53^+/+^ and p53^−/−^ HCT116 cells by using real-time PCR assays. The data are presented as mean±S.D. of three independent experiments. *P<0.05, **P<0.01

GOF mutant p53 proteins can transcriptionally regulate the expression of a large plethora of target genes and also transcriptionally regulate the expression of microRNAs, small non-coding RNAs that regulate gene expression at the posttranscriptional level.^18^ To search for the candidate miRNAs that could be regulated by p53, online tools, including miRWalk, miRanda, RNA22 and Targetscan, were used. Several miRNAs were proposed, among which seven of them were reported to be related to p53: miR-140, miR-30b, miR-3151, miR-506, miR-124, miR-30c, and miR-663b^19, 20, 21, 22, 23, 24^ (Figure 2a). The expression levels of these miRNAs were determined in p53^wt^, p53^mut^, p53^+/+^ and p53^−/−^ cells by using real-time PCR assays. In p53^mut^ cell line HT29, the expression levels were significantly downregulated except miR-3151 and miR-663b (which were significantly upregulated), compared with p53^wt^ cell line RKO (Figure 2b). Similar results were observed in p53^+/+^ and p53^−/−^ cells (Figure 2c): the expression levels of miR-3151 and miR-663b were upregulated in p53^−/−^ cells, while the expression levels of miR-140, miR-30b, miR-506, miR-124 and miR-30c were downregulated in p53^−/−^ cells compared with that in p53^+/+^ cells. Among the five downregulated miRNAs, miR-124 showed to be the most strongly downregulated in p53^mut^ and p53^−/−^ cells. These data indicated that these five miRNAs could be inhibited after p53 mutant or knocked out, and miR-124 was the most strongly suppressed one.

### The effect of PDT on miR-124 and iASPP mRNA expression

**Figure 3:**
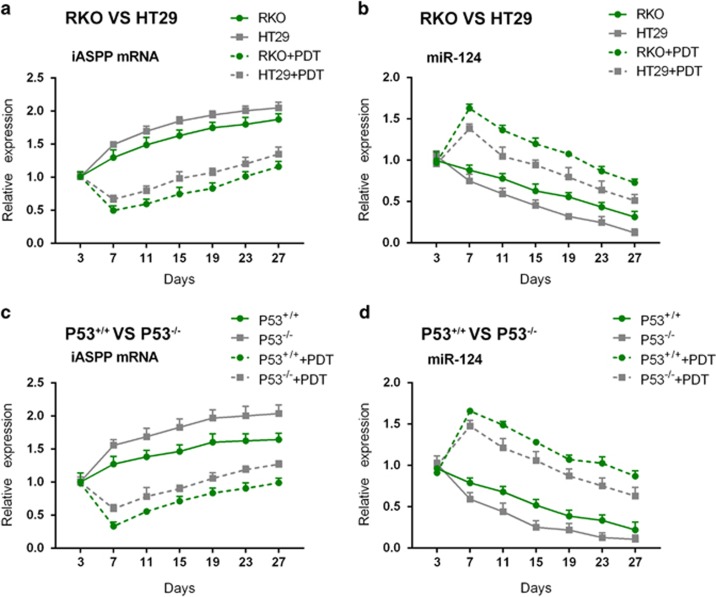
The effect of PDT on miR-124 and iASPP mRNA expression. (**a** and **c**) iASPP mRNA expression in HT29-, RKO-, p53^+/+^- and p53^−/−^ cell-derived tumors in the presence or absence of PDT was determined using real-time PCR on days 3, 7, 11, 15, 19, 23 and 27. (**b** and **d**) miR-124 expression in HT29-, RKO-, p53^+/+^- and p53^−/−^ cell-derived tumors in the presence or absence of PDT was determined using real-time PCR on days 3, 7, 11, 15, 19, 23 and 27. The data are presented as mean±S.D. of three independent experiments

We revealed that the expression levels of miR-124 and iASPP were altered in a p53 content-dependent manner in tumor tissues, and then we further investigated the effect of PDT on miR-124 and iASPP expression in mice tumors that were derived from different tumor cells. Expression levels of iASPP mRNA were upregulated in a time-dependent manner in all kinds of mice tumors (p53^wt^ (RKO) -, p53^mut^ (HT29) -, p53^+/+^- or p53^−/−^-derived mice tumors) without PDT treatment (Figures 3a and c); under PDT treatment, the expression of iASPP mRNA was downregulated to a valley value on day 7 by PDT treatment and returned gradually to higher levels at later time points; however, the expression levels of iASPP were lower in the PDT treatment groups than that in the non-PDT treatment groups (Figures 3a and c). In the absence of PDT treatment, miR-124 expression was downregulated in a time-dependent manner in all kinds of mice tumors (p53^wt^ (RKO) -, p53^mut^ (HT29) -, p53^+/+^- or p53^−/−^-derived mice tumors) (Figures 3b and d); in the presence of PDT treatment, miR-124 expression was upregulated to a peak value on day 7 by PDT treatment, and fell down gradually to lower levels at later time points; however, miR-124 expression in the PDT treatment groups was higher than that in the non-PDT treatment groups (Figures 3b and d).

### The influence of miR-124 or iASPP on p53^wt^/p53^mut^and p53^+/+^/p53^−/−^cells

**Figure 4:**
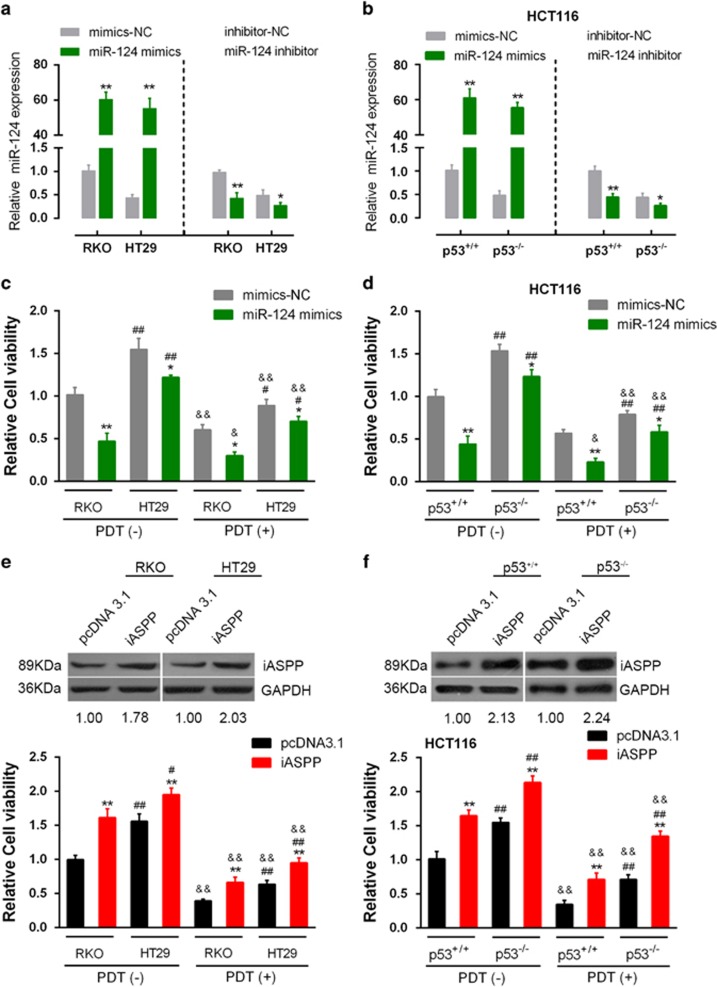
The influence of miR-124 or iASPP on p53^wt^/p53^mut^ and p53^+/+^/p53^−/−^ cells in response to PDT treatment. (**a**) and (**b**) miR-124 mimics or miR-124 inhibitor was transfected into RKO (p53^wt^), HT29 (p53^mut^), p53^+/+^ or p53^−/−^ cells to achieve miR-124 overexpression or miR-124 inhibition. The transfection efficiency was verified by using real-time PCR assays. (**c**) The viability of p53^wt^ CRC cell line RKO and p53^mut^ cell line HT 29 transfected with miR-SCR or miR-124 in the absence or presence of PDT was determined by using CCK-8 assays. (**d**) The viability of p53^+/+^ CRC cell line and p53^−/−^ cell line transfected with miR-SCR or miR-124 in the absence or presence of PDT was determined by using CCK-8 assays. (**e**) The viability of p53^wt^ CRC cell line RKO and p53^mut^ cell line HT 29 transfected with pcDNA3.1 or pcDNA3.1-iASPP in the absence or presence of PDT was determined by using CCK-8 assays. (**f**) The viability of p53^+/+^ CRC cell line and p53^−/−^ cell line transfected with pcDNA3.1 or pcDNA3.1-iASPP in the absence or presence of PDT was determined by using CCK-8 assays. The data are presented as mean±S.D. of three independent experiments. *P<0.05, **P<0.01, miR-124 mimics *versus* mimics-NC, or iASPP *versus* pcDNA3.1; #*P*<0.05, ##*P*<0.01, HT29 *versus* RKO, or p53^−/−^ HCT116 *versus* p53^+/+^ HCT116; &*P*<0.05, &&*P*<0.01, PDT (+) group *versus* PDT (−) group

Next we investigate the effect of miR-124 on the viability of p53^wt^/p53^mut^ CRC cells in response to PDT treatment. miR-124 mimics or miR-124 inhibitor was transfected into RKO, HT29, p53^+/+^ and p53^−/−^ HCT116 cells to achieve miR-124 overexpression or miR-124 inhibition (Figure 4a). Results showed that the viability of p53^wt^ CRC cell line RKO in the PDT (+)+miR-124 group was the lowest, whereas the viability of the p53mut CRC cell line HT29 in the PDT (−)+miR-SCR group was the highest (Figure 4b). Similar results were observed in p53^+/+^/p53^−/−^ cells: the viability of p53^+/+^CRC cell line RKO in the PDT (+)+miR-124 group was the lowest, whereas the viability of p53^−/−^CRC cell line HT29 in the PDT (−)+miR-SCR group was the highest (Figure 4c). The data suggested that miR-124 could promote the sensitivity of CRC cells to PDT treatment, so as to promote the inhibitory effect of PDT on CRC cells. Moreover, p53 mutation might reduce miR-124-induced sensitivity of CRC to PDT treatment.

Next we investigated the effect of iASPP on the viability of p53^wt^/p53^mut^ CRC cells in the absence or presence of PDT treatment. PcDNA3.1/iASPP vector was transfected into RKO, HT29, p53^+/+^ and p53^−/−^ HCT116 cells to achieve forced iASPP expression. The transfection efficiency was verified by using western blotting assays. Results showed that the viability of both p53^wt^ and p53^mut^ CRC cell line was significantly upregulated by iASPP overexpression in the PDT (−) group, more strongly promoted in p53^mut^ CRC cell line. The viability of both p53^wt^ and p53^mut^ CRC cell line was downregulated by PDT treatment and partially restored by iASPP overexpression. Among the two CRC cell lines, the viability of p53^wt^ CRC cells was more strongly suppressed by PDT treatment, while the viability of p53^mut^ CRC cells was more strongly restored by iASPP overexpression (Figure 4d). Similar results were observed in p53^+/+^/p53^−/−^ HCT116 cells: the viability of p53^+/+^ HCT116 cells in the absence of iASPP in the PDT (+) group was the lowest, whereas the viability of p53^−/−^ HCT116 cells in the presence of iASPP in the PDT (−) group was the highest (Figure 4e). The data suggested that iASPP could reduce the sensitivity of CRC cells to PDT treatment, thus reducing the inhibitory effect of PDT on CRC cells. Moreover, p53 mutation or knockout could amplify the effect of iASPP on CRC cells’ sensitivity to PDT.

### p53 regulates miR-124 expression by binding to its promoter

**Figure 5:**
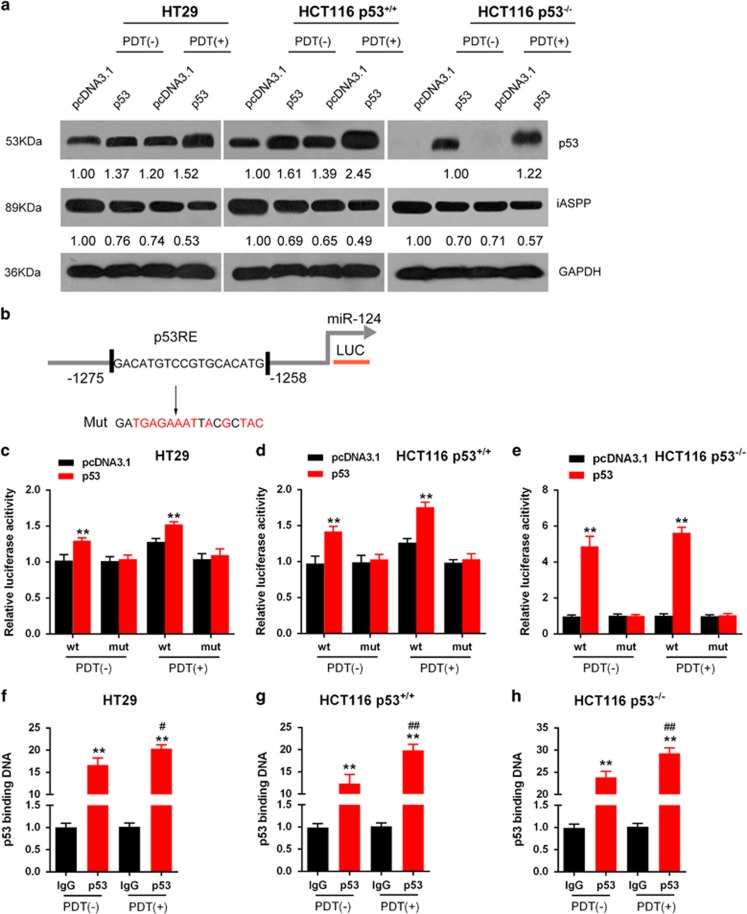
p53 regulates miR-124 expression by binding its promoter. (**a**) pcDNA3.1/p53 vector was transfected into HT29, p53^+/+^ HCT116 cell and p53^−/−^ HCT116 cell to achieve forced p53 expression in the presence or absence of PDT. (**b**) A schematic diagram of a potential p53 binding element in the promoter region of the miR-124 gene predicted by Jaspar database. A wt-miR-124 promoter luciferase reporter vector and a mut-miR-124 promoter luciferase reporter vector were constructed. (**c**–**e**) The indicated luciferase reporter vectors were co-transfected into HT29, p53^+/+^ HCT116 cells and p53^−/−^ HCT116 cells with pcDNA3.1/p53 vector in the presence or absence of PDT. The luciferase activity was then determined by using dual luciferase assays. (**f**–**h**) The real-time ChIP assay showed that the level of p53 antibody binding to miR-124 promoter was much greater than that of IgG in the presence or absence of PDT. The data are presented as mean±S.D. of three independent experiments. **P<0.01, compared with the pcDNA3.1 group; ^#^*P*<0.05, ^##^*P*<0.01, compared with the p53+PDT (–) group

To investigate the role of miR-124 further in CRC cells, we focused on the mechanism of miR-124 overexpression in these cells. pcDNA3.1/p53 was transfected into HT29, p53^+/+^ HCT116 cells and p53^−/−^ HCT116 cells to achieve forced p53 expression in the presence or absence of PDT treatment. p53 protein levels were then determined using western blotting assays. As shown in Figure 5a, p53 protein was increased in all p53-transfected cells, compared with that in pcDNA3.1-transfected cells; more importantly, p53 protein levels were higher in the PDT treatment group, compared with those in the non-PDT treatment group. Figure 5b is a schematic diagram of the potential p53-binding element in the promoter region of the miR-124 gene predicted by Jaspar database. A mutated binding element was constructed by mutating 13 bp within the binding element. We subcloned the wt- or mut-binding element and co-transfected the constructs with pcDNA3.1/p53 luciferase reporter gene vectors into HT29, p53^+/+^ HCT116 cells and p53^−/−^ HCT116 cells in the presence or absence of PDT treatment (Figures 5c–e). As shown in Figures 5c–e, p53 significantly promoted the luciferase activity as compared with pcDNA3.1 when co-transfected with wt-binding element either in the presence or absence of PDT treatment; in all cells, the luciferase activity was slightly stronger in the PDT treatment group, compared with that in the non-PDT treatment group, suggesting a PDT-promoted p53 protein level (Figure 5d). When the binding element was mutated, luciferase activity was not changed either in the presence or absence of PDT treatment, compared with that of the pcNDA3.1 (Figures 5c–e). Furthermore, the real-time chromatin immunoprecipitation (ChIP) assay showed that the level of p53 antibody binding to miR-124-binding element in the miR-124 promoter was much greater than that of IgG in all the three indicated cell lines (Figures 5f–h), suggesting that p53 might bind to the promoter of miR-124 to activate its expression. Similarly, in all cells, the level of p53 antibody binding to miR-124-binding element in miR-124 promoter was significantly promoted by PDT treatment (#*P*<0.05, ##*P*<0.01), compared with that in the absence of PDT, indicating that PDT treatment significantly upregulated p53 protein level (Figures 5f–h).

### The correlation of p53, miR-124 and iASPP expression in CRC tissues

**Figure 6:**
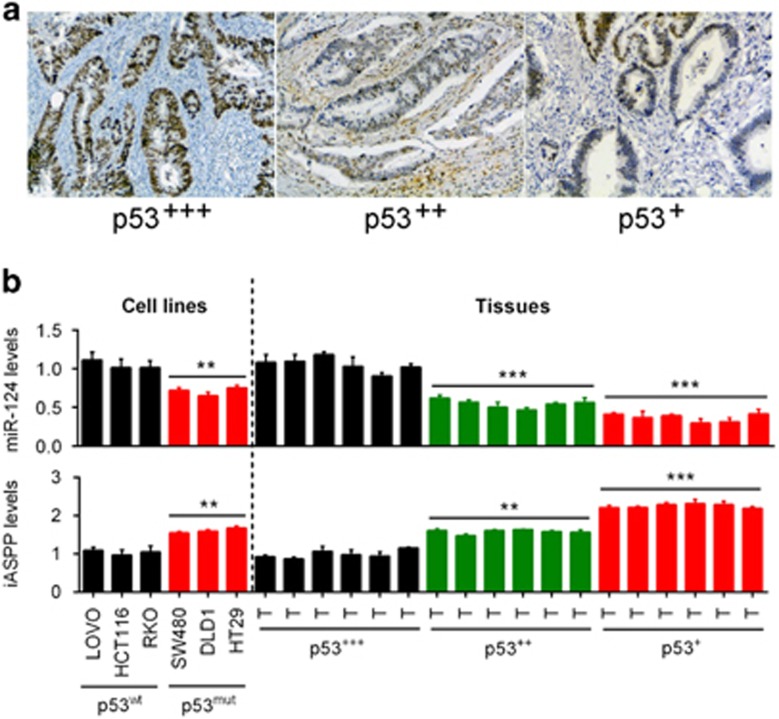
The correlation of p53, miR-124 and iASPP expression in CRC tissues. (**a**) Clinic samples containing diverse p53 levels have been obtained and the pathological states have been assessed. Results showed that the samples possessing higher p53 content had a better pathological condition. (**b**) miR-124 expression was upregulated in p53wt cells but downregulated in p53mut cells, and the expression levels were altered in a p53 content-dependent manner; iASPP expression was downregulated in p53wt cells but upregulated in p53mut cells, and the expression levels were altered in a p53 content-dependent manner. The data are presented as mean±S.D. of three independent experiments. **P<0.01, ***P<0.005

Clinic samples containing diverse p53 levels have been obtained and the pathological states have been assessed. Results showed that the samples possessing higher p53 content had a better pathological condition (Figure 6a). Our previous study revealed that miR-124 regulates the proliferation of CRC cells by targeting iASPP.^14^ To investigate the effect of mutant p53 on the expression of miR-124 and iASPP, we then determined miR-124 and iASPP expression in p53^wt^ cells and p53^mut^ cells. Results showed that miR-124 expression was upregulated in p53^wt^ cells but downregulated in p53^mut^ cells, and the expression levels were altered in a p53 content-dependent manner in tumor tissues; iASPP expression was downregulated in p53^wt^ cells but upregulated in p53^mut^ cells, and the expression levels were altered in a p53 content-dependent manner in tumor tissues (Figure 6b).

### Schematic diagram of the role of p53 mutation or knockout in PDT treatment

**Figure 7:**
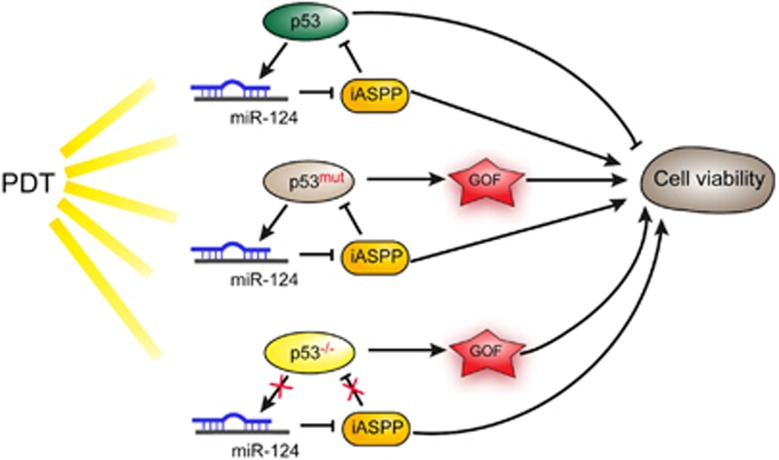
Schematic diagram of the role of p53 mutation or knockout in PDT treatment. In the present study, we revealed that p53 promoted miR-124 expression to inhibit iASPP expression, so as to amplify the inhibitory effect of PDT on CRC cell proliferation; after p53 mutation or knockout, miR-124 expression was downregulated while the iASPP expression was upregulated, so that the inhibitory effect of PDT on CRC cell proliferation was reduced

In the present study, we revealed that p53 promoted miR-124 expression to inhibit iASPP expression, so as to amplify the inhibitory effect of PDT on CRC cell proliferation; after p53 mutantion or knockout, miR-124 expression was downregulated while iASPP expression was upregulated, so that the inhibitory effect of PDT on CRC cell proliferation was reduced (Figure 7).

## Discussion

In recent years, PDT has been more and more applied clinically, the functional mechanism of which relies mainly on the generation of an active form of oxygen that may compromise cell viability and accelerate apoptosis.^25, 26, 27^ As a sensor of cellular stress, p53 is a relevant messenger of cell death signaling in PDT. The significant role of p53 in response to PDT has been reported for several clinically approved photosensitizers.^28, 29^ PDT induces an apoptotic pathway involving p53 and inactivates survival signal in human umbilical vein endothelial cells.^30^ Therefore, p53 mutation or deletion could weaken the sensitivity of cells to PDT. p53 mutation is commonly seen in the process of tumorigenesis and remains a tough obstacle to clinical treatment of malignancies. Most cases resistant to chemotherapy or radiotherapy display some extent of p53 abnormality.^31, 32, 33^ Mutant-type p53 cells were found to be significantly less sensitive to photofrin-mediated PDT.^13^ Wild-type p53 gene transfer into mutated p53 HT29 cells improves sensitivity to PDT via induction of apoptosis.^34^ p53 has been found involved in the above process as the mutation of p53 may influence the efficacy of PDT and make cancer cells less photosensitive to that PDT ignition.^35, 36^ In the present study, we observed that in p53-mutant or -deleted cells, p53 protein levels were significantly reduced, and the inhibitory effect of PDT on cell viability was significantly weakened, compared with wild-type p53 cells or p53^+/+^ cells. Also, tumor derived from p53-mutant or -deleted cells obtained continuously increased tumor size and caused shorter survival time. These findings revealed the major role of p53 mutation or deletion in PDT process; the killing effect of PDT on CRC cell might be p53 dependent.

According to previous studies, iASPP is a key inhibitor of the tumor-suppressor p53 in various cancers, including prostate cancer,^37^ liver cancer,^38^ lung cancer^39^ and glioma.^40^ Dong *et al.*^41^ summarize the oncogenic roles of iASPP in promoting proliferation, invasion, drug or radiation resistance and metastasis. The inhibition of iASPP phosphorylation with small molecules induces p53-dependent apoptosis and growth suppression.^41^ The role of iASPP in mediating p53-induced apoptosis and suppression of cell proliferation inspired us to validate its detailed function in PDT on CRC cells. We observed that exogenous iASPP expression increased the cell viability of CRC cell lines and attenuated the inhibitory effect of PDT on CRC cell lines. These suggested that iASPP might mediate p53-independent pathway in PDT process; in other words, as we have demonstrated that the killing effect of PDT on CRC cell might be p53 dependent, we further figure out that iASPP can suppress p53-induced cancer cell apoptosis under PDT treatment through inhibiting p53 expression, thus attenuating the killing effect of PDT on CRC cell.

Human cancer is associated with changes in miRNA expression. The pattern of miRNA expression varies dramatically across tumor types, and miRNA profiles reflect the developmental lineage and differentiation state of a tumor.^42^ miRNA is also likely to have critical roles in the PDT process.^43^ Hypoxia induced by PDT induces miR-210 expression, followed by an increased expression of both VEGF and miR-296.^44^ Bach *et al.*^45^ identified eight miRNAs that were significantly differentially expressed 5 h after PDT, compared with baseline levels, and an up to 15-fold transient upregulation of miR-634, -1246 and -1290 relative to their basal levels. Given the major role of p53 in PDT process, we screened out several candidate miRNAs that could be regulated by p53 and verified the expression levels of these miRNAs in p53^wt^, p53^mut^, p53^+/+^ and p53^−/−^ cells. Among all of the candidate miRNAs, miR-124 showed to be significantly downregulated in p53^mut^ and p53^−/−^ cells. In our previous study, we revealed that miR-124 could inhibit iASPP by direct targeting to suppress CRC cell proliferation so as to affect the course of CRC.^14^ Here we validated their functional roles in PDT on CRC cells. For the first time, we demonstrated the amplificatory effect of miR-124 on PDT suppressing CRC cell viability, which could be partially abolished by p53 mutation or deletion; on the contrary, iASPP suppressed the inhibitory effect of PDT on CRC cell viability, which could be amplified by p53 mutation or deletion. On day 7 after PDT, the expression level of miR-124 presented a peak value, while iASPP expression presented a valley value at the same time, which was consistent with the tumor size alternation trend in response to PDT. These all suggested the important role of miR-124/iASPP in PDT process.

The biological effects of p53 are largely due to its function as a transcriptional regulator.^46^ p53 induces the expression and/or maturation of several miRNAs, which leads to the repression of critical effector proteins. The frequent genetic and epigenetic alterations of p53-regulated miRNAs in tumors indicate that they have an important role in cancer initiation and/or progression.^47^ Jeong *et al.*^23^ characterized miR-124 promoter region and identified a functional p53-binding site. In agreement with this finding, endogenous or ectopic expression of wild-type p53 increased miR-124 levels.^23^ Interestingly, here we found that p53 binds to the promoter of miR-124 to promote its expression in the presence or absence of PDT and subsequently affect the expression of miR-124 downstream target gene, so as to form a regulation loop in the wild-type p53 cells. In p53-mutant or -deleted cells, this binding no longer worked to promote miR-124 expression, and iASPP expression increased, which finally resulted in attenuated killing effect of PDT on CRC cells and promoted CRC cell viability.

All in all, the interactive modulation among miR and iASPP in p53-mutant or -deleted cells may serve as a crucial pathway, which mediates therapy resistance when p53 is mutated or deleted, in the process of PDT treatment of CRC. These findings may well be meaningful in bringing up the efficacy and optimizing the strategy of PDT. Nevertheless, considering the firm association between p53 mutation and tumor metastasis and resistance to chemotherapy or radiotherapy, the newly found miR–iASPP interaction may well enlighten us with novel directions in dealing with these problems in the near future.

## Materials and methods

### Photosensitizers

5,10,15,20-tetrakis(pentafluorophenyl)-2,3-(methano[Nmethyl] iminomethano])chlorin) (H2TFPC) and H2TFPCSGlc (Figure 1a) were synthesized and provided by the laboratory of the Kyoto University (Kyoto, Japan) and Okayama University of Science (Okayama, Japan). They contain no isomers, based on 1H-NMRand 19F-NMR measurements.

### Cell culture

HCT116, LoVo, RKO, HT29, DLD1 and SW480 cell lines have been characterized in detail and was provided by ATCC (Manassas, VA, USA). These were cultured in Eagle minimum essential medium (Wako Pure Chemical Industries Co. Ltd., Wako, Japan) supplemented with 10% FBS and 1% ampicillin and streptomycin under 5% CO_2_ at 37 °C.

### PDT and phototoxicity experiments

MTHPC was obtained from Biolitec AG (Jena, Germany) at a concentration of 5.88 mM (4 mg/ml diluted with the ethanol/propylene glycol solvent to a working stock concentration of 0.588 mM (400 *μ*g/ml)) and stored at 4 °C in the dark. Porfimersodium was obtained from Seehof Laboratorium (Wesselburenerkoog, Germany) at a concentration of 9.96 mg/ml (corresponding to 16.67 mM based on a nominal molecular weight of 600 g/mol) and stored at 4 °C in the dark. Resazurin sodium salt was obtained from Sigma-Aldrich (Vienna, Austria). Cells cultured in 96-well microplates (black walls, clear bottom) were washed once with 100 *μ*l sf-DMEM and incubated with 0.588 *μ*M (400 ng/ml) mTHPC and 1.2 *μ*g/ml (2 *μ*M) porfimer sodium, respectively, again using 100 *μ*l sf-DMEM. Subsequent to 20 h incubation, photosensitized cells were illuminated from below the microplates using LED arrays as light sources.^48^ 660 nm (0.65 J/cm^2^) and 624 nm (4.30 J/cm^2^) dominant wavelength with a current of 5 A were employed for 3 or 7 min (for mTHPC and porfimer sodium, respectively, described in). Directly after illumination and 24 h afterwards, the cellular viability was assessed by adding 20 *μ*l 2.5 mM resazurinin PBS to each well, incubating for 2 h at 37 °C and measured using the Infinite M200 microplate reader (Tecan, Grödig, Austria) at *λ*_ex_=535 nm and *λ*_em_=588 nm. Similar to other viability assays (CCK-8), the fluorimetric resazurin method indicates the proportion of metabolically active (viable) cells. For the current data set (mTHPC and porfimer), the obtained values were corrected for blank wells and related to the baseline viability (0 h values); the second data set (mTHPC, adopted from an earlier publication) was related to untreated controls measured at 24 h after illumination. For correlation of miR expression data with the phototoxicity after correction by the cell-dependent uptake of the PS, the fluorimetry uptake values were adopted from the mentioned earlier study. For the current set of mTHPC- and porfimer sodium-based phototoxicity, the cellular uptake (corrected by total protein) was measured by fluorimertry as described previously.

### Mouse experiments

HT29, RKO, p53^+/+^ HCT116 and p53^−/−^ HCT116 cell lines were used for the injection of the mice, 10 mice in each group. Cells (5 × 10^5^ cells per animal) were suspended in 100 *μ*l of serum-free DMEM mixed with 100 *μ*l of 10% Matrigel (BD, Franklin Lakes, NJ, USA) and injected subcutaneously into 5–8-week-old female BALB/c nude mice. Tumor size was measured every 2 days from day 3 to day 27 in the absence or presence of PDT treatment. Animals were humanely killed depending on tumor volume. Expression levels of miR-124 and iASPP was determined in tumors that were derived from the indicated cells by injection on days 3, 7, 13, 20 and 27. The tumor was snap-frozen, and RNA was extracted for measurement of miR-124 and iASPP levels by specific Taqman probes. The methods were carried out in accordance with the relevant guidelines and regulations. Animal protocols were approved by the Ethics Committee of the Second Xiangya Hospital, Central South University.

### RNA extraction and SYBR Green quantitative PCR analysis

Total RNA was extracted from CRC cells using TRIzol reagent (Invitrogen, Carlsbad, CA, USA). The expression levels of miRNAs were detected using the Hairpin-it miRNAs qPCR Kit (Genepharma, Shanghai, China). The expression of RNU6B served as an endogenous control. iASPP expression was measured using a SYBR Green qPCR assay (Takara, Dalian, China). The data were processed according to the 2^−ΔΔCT^ method; the relative expression of miR-124 was calculated with the formula, 2^−(CTmiRNA−CTRNU6B)^. The primers are shown in Supplementary Table 1.

### CCK-8 cell proliferation assay

Cell proliferation rates were measured using the CCK-8 (Beyotime, Hangzhou, China) methods. Approximately 0.5 × 10^4^ cells were seeded in each 96-well plate for 24 h, after which they were transfected with the indicated miRNA or siRNA and further incubated for 24 or 48 h. CCK-8 reagent (10 *μ*l) was added to each well 1 h before the end point of each incubation period. The OD_450_ value was determined for each well using a microplate reader. The relative cell viability was normalized to the value of RKO in the absence of PDT 24 h after transfection.

### Luciferase reporter assay

Cells were transfected with miR-124 and pGL3 luciferase reporter constructs harboring the miR-124 target sequence. After 24 h, the activities of firefly luciferase and Renilla luciferase were measured in the cell lysates using a Dual-Luciferase Assay System (Promega, Madison, WI, USA). For the luciferase transcription reporter assay, miR-124 gene promoter sequences (WT or site deletion) were cloned into the promoter region of the pGL3-Basic vector, and luciferase activity was measured as described above.

### Western blotting analysis

The protein levels of iASPP and p53 in CRC cells were detected by performing immunoblotting. We lysed cultured or transfected cells in RIPA buffer with 1% PMSF and loaded protein onto a SDS-PAGE minigel and transferred them onto PVDF membrane. After probing with the following antibodies: iASPP (ab115605, Abcam, Cambridge, MA, USA), p53 (ab1431, Abcam) and GAPDH (ab9484, Abcam) at 4 °C overnight, the blots were subsequently incubated with HRP-conjugated secondary antibody (1 : 5000). ECL substrates was used to visualize signals (Millipore, Danvers, MA, USA). GAPDH was used as an endogenous protein for normalization.

### Chromatin immunoprecipitation

Briefly, the treated cells were cross-linked with 1% formaldehyde, sheared to an average size of 400 bp DNA and immunoprecipitated using antibodies against p53 (anti-p53, clone DO-7, Sigma-Aldrich). The ChIP-PCR primers were designed to amplify the promoter regions containing putative p53-binding sites within miR-124, as previously described (MIR124-p53BS-F and MIR155-p53BS-R are used in the ChIP assay, shown in Supplementary Table S1). A positive control antibody (RNA polymerase II) and a negative control non-immune IgG were used to demonstrate the efficacy of the kit reagents (Epigentek Group Inc., Framingdale, NY, USA, P-2025-48). The immunoprecipitated DNA was subsequently cleaned, released and eluted. The eluted DNA was used for downstream applications, such as ChIP-PCR. The fold-enrichment (FE) was calculated as the ratio of the amplification efficiency of the ChIP sample to that of the non-immune IgG. The amplification efficiency of RNA Polymerase II was used as a positive control. FE%=2 (IgG CT−Sample CT) × 100%.

### Immunohistochemistry

Immunohistochemical (IHC) profile of the tumor was assessed by subjecting one section each from a block to p53 immunostain. IHC was performed on 4 *μ*m-thick sections from 10% formalin-fixed paraffin-embedded specimens, according to the streptoavidin–biotin immunoperoxidase technique (Dako-cytomation, Santa Clara, CA, USA). Multiple slides were evaluated, and ideal section was used for IHC staining.

These sections were reviewed by three blind-folded pathologists; strong brown nuclear immunoreactivity was considered as positive staining; a semiquantitative scoring system was employed to assess the level of p53 reactivity: 0 – was assigned when no staining was observed, 1 – when <10% of tumor cell nuclei were reactive, 2 – when >10%, but <33% of the nuclei stained, and 3 – if >33% of nuclei were positive.^49, 50, 51^ Herein, a score of 0–1 was defined as p53+, 2 was defined as p53++, 3 was defined as p53+++.

### Statistical analyses

Data from three independent experiments were expressed as mean±S.D. and processed using the SPSS17.0 statistical software (IBM, Armonk, NY, USA). Differences between two groups were compared using Student’s paired test. Differences among the groups in the above assays were estimated using one-way ANOVA. A *P*-value <0.05 was considered significant.

## Publisher’s Note:

Springer Nature remains neutral with regard to jurisdictional claims in published maps and institutional affiliations.
