# Supplementary material for: Wild-type and mutant p53 differentially modulate miR-124/iASPP feedback following pohotodynamic therapy in human colon cancer cell line
Source: Cell Death Dis. 2017 Oct 12;8(10):e3096–. doi: 10.1038/cddis.2017.477 (PMC5682646; doi:10.1038/cddis.2017.477)
Supplement: Supplementary Table 1 [file cddis2017477x1.docx]

Table 1 Oligonucleotides

| Primer | Sequence |
| --- | --- |
| iASPP realtime forward | GGCGGTGAAGGAGATGAAC |
| iASPP realtime reverse | TGATGAGGAAATCCACGATAGAG |
| miR-140-5p-RT | GTCGTATCCAGTGCAGGGTCCGAGGTATTCGCACTGGATACGACCTACCT |
| miR-140-5p-F | GCACGGCAGTGGTTTTACCCTA |
| miR-30b-3p-RT | GTCGTATCCAGTGCAGGGTCCGAGGTATTCGCACTGGATACGACGAAGTA |
| miR-30b-3p-F | GCTAGGCTGGGAGGTGGATGTT |
| miR-3151-5p-RT | GTCGTATCCAGTGCAGGGTCCGAGGTATTCGCACTGGATACGACACCTGA |
| miR-3151-5p-F | GCTAATGGTGGGGCAATGGGA |
| miR-506-3p-RT | GTCGTATCCAGTGCAGGGTCCGAGGTATTCGCACTGGATACGACTCTACT |
| miR-506-3p-F | GCGACGTAAGGCACCCTTCTG |
| miR-124-3p-RT | GTCGTATCCAGTGCAGGGTCCGAGGTATTCGCACTGGATACGACGGCATT |
| miR-124-3p-F | GTCGCCTAAGGCACGCGGTG |
| miR-30c-RT | GTCGTATCCAGTGCAGGGTCCGAGGTATTCGCACTGGATACGACAGAGTA |
| miR-30c-F | GACGGTCTGGGAGAAGGCTGTT |
| miR-663b-RT | GTCGTATCCAGTGCAGGGTCCGAGGTATTCGCACTGGATACGACCCTCAG |
| miR-663b-F | ATGCATGGTGGCCCGGCCGTGC |
| Universal reverse primer | GTGCAGGGTCCGAGGT |
| U6-f | CTCGCTTCGGCAGCACA |
| U6-r | AACGCTTCACGAATTTGCGT |
| GAPDH realtime forward | CTCAGACGGCAGGTCAGGTCCACC |
| GAPDH realtime reverse | CCACCCATGGCAAATTCCATGGCA |
| MiR-124-promoter-F | AAAGCTAGCCAGTTCGGGCGTTGGCCGTGGC |
| MiR-124-promoter-R | TTTCTCGAGCAGCCCCATTCTTGGCATTCA |
| Mut-miR124-promoter-F | AGAAATTGATGAGAAATTACGCTACGATCATTGCGCCATGAGGCGGCGACAGGATTT |
| Mut-miR124-promoter-R | AAATCCTGTCGCCGCCTCATGGCGCAATGATCGTAGCGTAATTTCTCATCAATTTCT |
| MIR124-p53BS-F(for ChIP) | GGGTGTCTGTCGATAGGA |
| MIR124-p53BS-R(for ChIP) | TCTCCACACTGTTTACACA |
